# COVID-19 as a Catalyst for Same-Day Discharge Total Shoulder Arthroplasty

**DOI:** 10.3390/jcm10245908

**Published:** 2021-12-16

**Authors:** Mariano E. Menendez, Noah Keegan, Brian C. Werner, Patrick J. Denard

**Affiliations:** 1 Oregon Shoulder Institute at Southern Oregon Orthopedics, Medford, OR 97504, USA; marianofurrer@gmail.com (M.E.M.); noahtkeegan@gmail.com (N.K.); 2 Department of Orthopaedic Surgery, University of Virginia, Charlottesville, VA 22903, USA; BCW4X@hscmail.mcc.virginia.edu

**Keywords:** shoulder arthroplasty, COVID, coronavirus, length of stay, same-day discharge, pandemic

## Abstract

The COVID-19 pandemic caused major disruptions to the healthcare system, but its impact on the transition to same-day discharge shoulder arthroplasty remains unexplored. This study assessed the effect of COVID-19 on length of stay (LOS), same-day discharge rates, and other markers of resource use after elective total shoulder arthroplasty. A total of 508 consecutive patients undergoing elective primary total shoulder arthroplasty between 2019 and 2021 were identified and divided into 2 cohorts: “pre-COVID” (March 2019–March 2020; *n* = 263) and “post-COVID” (May 2020–March 2021; *n* = 245). No elective shoulder arthroplasties were performed at our practice between 18 March and 11 May 2020. Outcome measures included LOS, same-day discharge, discharge location, and 90-day emergency department (ED) visits, readmissions and reoperations. There were no significant differences in baseline preoperative patient characteristics. Shoulder arthroplasty performed post-COVID was associated with a shorter LOS (12 vs. 16 h, *p* = 0.017) and a higher rate of same-day discharge (87.3 vs. 79.1%, *p* = 0.013). The rate of discharge to skilled nursing facilities was similarly low between the groups (1.9 vs. 2.0%, *p* = 0.915). There was a significant reduction in the rate of 90-day ED visits post-COVID (7.4 vs. 13.3%, *p* = 0.029), while there were no differences in 90-day reoperation (2.0 vs. 1.5%, *p* = 0.745) or readmission rates (1.2 vs. 1.9%, *p* = 0.724). The COVID-19 pandemic seems to have accelerated the shift towards shorter stays and more same-day discharge shoulder arthroplasties, while reducing unexpected acute health needs (e.g., ED visits) without adversely affecting readmission and reoperation rates.

## 1. Introduction

The coronavirus (COVID-19) pandemic caused drastic disruptions to the provision of elective orthopedic surgery services in the United States [1]. This crisis also presented an opportunity for value optimization by promoting collaboration and creative thinking. One well-documented disruptive change has been the swift adoption of telehealth services [2,3]. The COVID outbreak may have also catalyzed the shift towards more resource-efficient outpatient joint arthroplasty, but this remains speculation [4].

Elective shoulder arthroplasty is an increasingly popular and highly standardized procedure that has been classically performed as inpatient [5,6,7]. It is unclear whether COVID has changed any of this. Patients may now be more inclined to go home shortly after surgery to minimize risk of contagion [8], and to rely more on technology (e.g., email, telehealth) to address postoperative concerns that would traditionally warrant a visit to the emergency department (ED). Hospitals may also be incentivized to more expeditiously discharge elective surgery patients to ensure continued bed capacity for potential COVID surges.

This study sought to determine the impact of the COVID-19 pandemic on length of stay (LOS) and same-day discharge rates after elective total shoulder arthroplasty. Additionally, we examined discharge disposition patterns, ED visits, readmissions and reoperations. The hypothesis was that LOS decreased post-COVID, despite no change in patient characteristics.

## 2. Methods

### 2.1. Study Design

A retrospective study was conducted of a consecutive series of shoulder arthroplasties performed at a single private practice institution. Institutional review board approval was obtained for this study. Our registry was queried to identify all patients who underwent elective primary total shoulder arthroplasty (anatomic (ATSA) or reverse (RTSA)) between March 2019 and March 2021 by a single fellowship-trained shoulder surgeon. The inclusion criteria were: (1) an ATSA or RTSA and (2) minimum follow-up of 90 days. To achieve a homogenous sample of patients at low surgical risk, an a priori decision was made to exclude patients whose indication for surgery was traumatic, and those undergoing revision surgery.

Following the 18 March 2020 recommendation by the Centers for Medicare and Medicaid Services to postpone non-essential surgeries in response to the COVID-19 virus, no elective shoulder arthroplasties were performed at our practice until 11 May 2020. As such, the study sample was divided into two cohorts: the “pre-COVID” group for surgeries performed before 18 March 2020, and the “post-COVID” group for cases performed on or after 11 May 2020. Notably, the treating surgeon had nearly 10 years of experience at the beginning of the study period. During the study period there was no change in postoperative protocols or in the design of the implants used by the primary surgeon.

### 2.2. Outcomes Measures and Explanatory Variables

The main outcomes of interest included LOS (measured in hours after surgery) and same-day discharge. Discharge disposition (home versus skilled nursing facility (SNF)) was also recorded. Electronic medical records linked to the local hospital were reviewed to collect data on ED visits, readmissions, and reoperations within 90 days of surgery.

Several patient characteristics that might affect the influence of the COVID-19 pandemic on resource allocation after shoulder arthroplasty were recorded. Specifically, data were collected on age, sex, body mass index (BMI), and the presence of co-morbidities including diabetes, chronic obstructive pulmonary disease (COPD), and tobacco use. Surgical location (hospital versus ambulatory surgery center) data were also collected.

### 2.3. Statistical Analysis

To compare both baseline patient characteristics and postoperative outcomes between the pre- and post-COVID cohorts, Pearson chi-square tests were used for categorical variables and independent samples T-tests were used for continuous variables. Continuous variables were presented in terms of the mean and standard deviation (SD), and categorical variables were reported with frequencies and percentages. Statistical tests were 2-sided with *p* < 0.05 denoting statistical significance.

## 3. Results

A total of 508 patients met the study criteria. The study population consisted of 241 (47%) women and 267 men, with a mean (SD) age of 71 (8) years and BMI of 30 (6). Overall, 63% of patients underwent RTSA, while the remaining 37% had ATSA. There were no significant differences in any of the baseline patient characteristics between the pre- and post-COVID groups (Table 1). There was no difference in the rate of procedures performed in the hospital versus surgery center setting between the two groups (Table 1).

**Figure 1:**
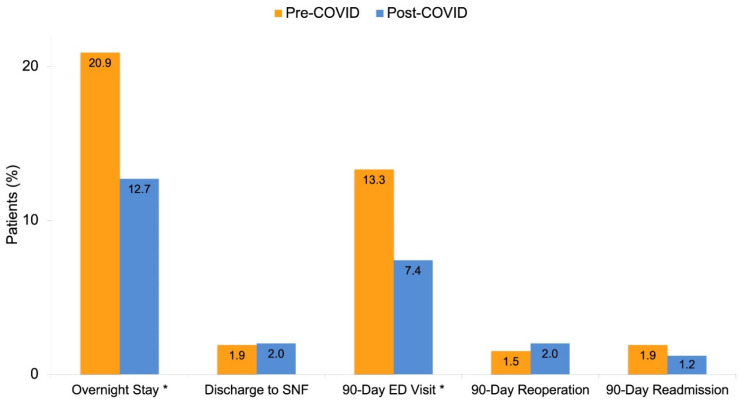
Outcomes after total shoulder arthroplasty in the pre- and post-COVID groups. Asterisks denote statistical significance (*p* < 0.05).

**Figure 2:**
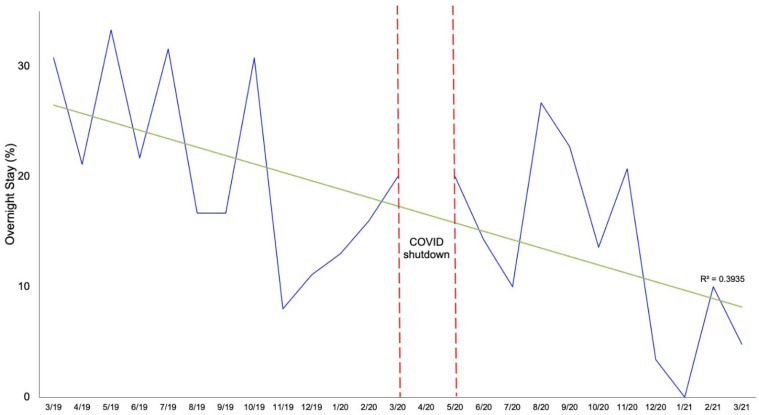
Rate of shoulder arthroplasties requiring overnight hospital stays over time.

Shoulder arthroplasty performed in the post-COVID cohort was associated with a shorter LOS (12 vs. 16 h, *p* = 0.017) and higher rate of same-day discharge to home (87.3 vs. 79.1%; Figure 1, Table 2). Figure 2 is a more granular representation of the decline in the proportion of surgeries with overnight stays.

The rate of discharge to SNFs was similar between the groups (1.9 vs. 2.0%, *p* = 0.915). There was a significant reduction in the rate of 90-day ED visits in the post-COVID cohort (7.4 vs. 13.3%, *p* = 0.029), while there was no difference with regard to 90-day reoperation (2.0 vs. 1.5%, *p* = 0.745) and readmission rates (1.2 vs. 1.9%, *p* = 0.724; Table 2).

## 4. Discussion

The COVID-19 outbreak upended traditional health system practices amid an environment that demanded an accelerated pace of innovation. Health systems were faced with difficult decisions as to how to safely resume margin-producing elective orthopedic surgery in the midst of the pandemic. Many have suggested transitioning more joint arthroplasty procedures to the outpatient setting [4,9,10], but whether this actually has taken place is unclear. This study showed that shoulder arthroplasty following the resumption of elective surgery during the COVID-19 pandemic was associated with a shorter LOS and higher rate of same-day discharge.

The finding that baseline preoperative patient characteristics remained unchanged compared to before the outbreak suggests that the observed changes in discharge patterns may indeed be a direct consequence of COVID. There is recent evidence that sociodemographic and psychological factors may have more influence than patient infirmity and technical issues in the variation in LOS and discharge disposition after shoulder arthroplasty [11]. Although this requires formal investigation, it is possible that patients may be more motivated to go home after surgery during the pandemic to minimize risk of contagion [8]. Indeed, it has been our experience during the pandemic that patients are more invested in making arrangements for going home the same day of surgery. Health systems may also be pushing for early elective surgery discharges to limit exposure and reallocate resources to sicker patients [9].

The observation that 90-day readmissions, reoperations, and ED visits did not increase following the resumption of elective surgery during the pandemic is reassuring. This is consistent with the growing realization that shorter postoperative stays after shoulder arthroplasty are safe. Shorter LOS and/or same-day discharge following shoulder arthroplasty do not seem to increase the risk of postoperative mortality and morbidity [12,13]. The important addition of the current study to this literature is the fact that both cohorts represented the majority of the shoulder arthroplasty population in the surgeon’s practice. The near 90% utilization of same day discharge in the post-COVID cohort indicates that there was limited potential for patient selection bias. In other words, outpatient shoulder arthroplasty is safe in not only selected patients, but in the majority of cases based on the findings of the current study.

Interestingly, we found that the rate of ED visits decreased significantly from 13.3% (pre-pandemic) to 7.4%. It may be that patients are now more likely to use and rely on technology (e.g., emails with image exchange, telehealth) to address postoperative concerns that would traditionally warrant a visit to the ED. The observed reduction in ED visits may indicate that some of them are preventable with the use of technology and improved postoperative care coordination. This subject deserves further study. Although another explanation could be that patients were more fearful of postoperative ED visits, this is not supported by the lack of change in the 90-day complication or re-operation rate.

The principal strengths of our study include its relatively large sample size and the fact that all procedures were performed by the same experienced surgeon, thus reducing surgeon variation in perioperative protocols. Nonetheless, our analysis was subject to several shortcomings that might be addressed in future research. First, the retrospective nature of this study does not allow causal inference. Therefore, we can only determine associations between COVID and the outcomes of interest. Second, because this study was performed at a private practice with a high pre-COVID rate of same day discharge shoulder arthroplasty, the results may lack generalizability. However, one might expect an even greater increase in the rate of outpatient shoulder arthroplasty among practices with traditionally higher rates of inpatient procedures. Future studies should evaluate and compare shoulder arthroplasty discharge patterns pre- and post-COVID across different practices and regions. Third, while we collected data on multiple markers of postoperative resource use (e.g., LOS, discharge disposition, ED visits, readmissions, reoperations), we did not assess patient experience and functional outcomes to better define the value equation. Fourth, there was a trend towards a potentially clinically relevant (+5.5% difference) higher rate of diabetes in the pre-COVID cohort compared to the post-COVID cohort which, while not yet significant, may affect results in larger samples. Finally, our study was limited in follow-up duration (90 days) due to the recency of the pandemic.

## 5. Conclusions

This study provides evidence that the COVID-19 pandemic may have accelerated the shift towards shorter stays and more same-day discharge shoulder arthroplasties, while reducing unexpected acute health needs (e.g., ED visits) without adversely affecting readmission and reoperation rates. These findings may be generalizable to other discretionary orthopedic procedures. Additional research should evaluate and compare the patient experience and functional outcomes following elective shoulder arthroplasty before and during the pandemic.

## Figures and Tables

**Table 1:** Characteristics of the study population.

Parameter	All Patients	Period		*p*
		Pre-COVID	Post-COVID	
Total †	508 (100)	263 (51.8)	245 (48.2)	
Age * (year)	70.5 ± 8.2	70.7 ± 7.9	70.3 ± 8.6	0.603
Sex †				
Female	241 (47.4)	119 (45.2)	122 (49.8)	0.305
Male	267 (52.6)	144 (54.8)	123 (50.2)	
BMI *	30.2 ± 6.4	30.5 ± 6.3	29.9 ± 6.5	0.329
Diabetes †	83 (16.3)	50 (19.0)	33 (13.5)	0.091
Chronic obstructive pulmonary disease †	38 (7.5)	23 (8.7)	15 (6.1)	0.262
Tobacco use †	23 (4.5)	8 (3.0)	15 (6.1)	0.095
Total shoulder arthroplasty type †				
Anatomic	190 (37.4)	95 (36.1)	95 (38.8)	0.537
Reverse	318 (62.6)	168 (63.9)	150 (61.2)	
Surgical location †				
Hospital	409 (80.5)	213 (81.0)	196 (80.0)	0.779
Ambulatory surgery center	99 (19.5)	50 (19.0)	49 (20.0)	

BMI = body mass index. * The values are given as the mean and the standard deviation. † The values are given as the number of patients, with the percentage in parentheses.

**Table 2:** Outcomes after total shoulder arthroplasty.

Parameter	All Patients	Period		*p*
		Pre-COVID	Post-COVID	
Same-day discharge †	422 (83.1)	208 (79.1)	214 (87.3)	0.013
Length of stay * (hours)	14 (3 to 192)	16 (3 to 120)	12 (3 to 192)	0.017
Discharge to skilled nursing facility †	10 (2.0)	5 (1.9)	5 (2.0)	0.915
ED visit within 90 days of surgery †	53 (10.5)	35 (13.3)	18 (7.4)	0.029
Postoperative pain	7 (1.4)	4 (1.5)	3 (1.2)	-
Wound issue	10 (2.0)	7 (2.7)	3 (1.2)	-
Medical issue	28 (5.5)	21 (8.0)	7 (2.9)	-
Musculoskeletal trauma and injury	8 (1.6)	3 (1.1)	5 (2.0)	-
Reoperation within 90 days of surgery †	9 (1.8)	4 (1.5)	5 (2.0)	0.745
Readmission within 90 days of surgery †	8 (1.6)	5 (1.9)	3 (1.2)	0.724

ED = emergency department. * The values are given as the mean, with the range in parentheses. † The values are given as the number of patients, with the percentage in parentheses.

## Data Availability

Details regarding where data supporting reported results can be requested at the following e-mail address: pjdenard@gmail.com.
